# Correlation between exposure to fine particulate matter and hypertensive disorders of pregnancy in Shanghai, China

**DOI:** 10.1186/s12940-020-00655-1

**Published:** 2020-09-17

**Authors:** Xiujuan Su, Yan Zhao, Yingying Yang, Jing Hua

**Affiliations:** Department of Women & Children’s Health Care, Shanghai First Maternity and Infant Hospital, Tongji University School of Medicine, No.2699, West Gaoke Road, Shanghai, 200040 China

**Keywords:** Fine particulate matter (PM_2.5_), Hypertensive disorders of pregnancy, Hypertension, Parity, Relative excess risk due to interaction

## Abstract

**Background:**

Association between fine particulate matter (PM_2.5_) and hypertensive disorders of pregnancy (HDP) is inconsistent and appears to change in each trimester. We aim to investigate the association of exposure to ambient PM_2.5_ in early pregnancy with HDP.

**Methods:**

A retrospective cohort study was performed among 8776 women with singleton pregnancy who attended the antenatal clinic before 20 gestational weeks in a tertiary women’s hospital during 2014–2015. Land use regression models were used to predict individual levels of PM_2.5_ exposure.

**Results:**

The average PM_2.5_ concentration during the first 20 gestational weeks ranged from 28.6 to 74.8 μg m^− 3^ [median, 51.4 μg m^− 3^; interquartile range, 47.3–57.8 μg m^− 3^]. A total of 440 (5.0%) women was diagnosed with HDP. The restricted cubic spline showed a positive exposure-response relationship between the PM_2.5_ concentration and risk of HDP. We observed an association between PM_2.5_ exposure during the first trimester with HDP (RR = 3.89 per 10 μg m^− 3^, 95% CI: 1.45–10.43), but not during the second trimester (RR = 0.71 per 10 μg m^− 3^, 95% CI: 0.40–1.27). Compared with their counterparts, nulliparous women who were exposed to high levels of PM_2.5_ in the index pregnancy had a higher risk of developing HDP [the relative excess risk due to interaction was 0.92 (0.46–1.38)].

**Conclusion:**

Our findings suggest that PM_2.5_ exposure during the first trimester is associated with the development of HDP. The effect estimate is more obvious for nulliparous women than multiparous women.

## Background

Hypertensive disorders of pregnancy (HDP), including gestational hypertension, preeclampsia, and eclampsia, exert substantial adverse effects on both maternal and foetal health [1, 2]. It complicates up to 10% pregnancies [3, 4] and ranks as the second leading causes of maternal mortality in China [5]. Despite the serious consequences, the biological mechanisms underlying HDP remain unclear.

Fine particulate matter (median aerodynamic diameter ≤ 2.5 μm; PM_2.5_), has been proposed to be associated with the incidence of hypertension in the general population [6–8]. PM_2.5_ generally has been associated with increased risks of cardiovascular diseases within hours to days of exposure in susceptible individuals [9]. Due to cardiovascular changes that occur as part of a normal pregnancy, pregnant women might be more vulnerable to the adverse effects of PM_2.5_, while its association with HDP is inconsistent and appears to change in each trimester [10–13]. Two systematic reviews, both of which were published in 2014, reported contradictory effect estimates between HDP and PM_2.5_ exposure during pregnancy [14, 15]. A case-control study of 298 predominantly Hispanic women reported an association between PM_2.5_ exposure during the first trimester, but not the second trimester, and an increased risk of HDP among non-obese women [16]. Additionally, two other studies based on the registry data suggested an association between second-trimester exposure to PM_2.5_ and an increased risk of HDP [12, 17]. The inconsistent findings may be due to the differences in PM_2.5_ concentrations and the ethnicity of the population [13]. We are not aware of any studies that have evaluated the association between HDP and exposure to a higher concentration of PM_2.5_ than the WHO interim target-1 of PM_2.5_ (35 μg m^− 3^) [18].

In this retrospective cohort study, we sought to investigate the association between exposure to ambient PM_2.5_ in early pregnancy with HDP. We hypothesize that the risk of HDP increases as the PM_2.5_ concentration increases and varies according to the trimester of pregnancy.

## Methods

### Study population

We performed a hospital-based, retrospective cohort study among pregnant women with a singleton pregnancy, who were attending the antenatal clinic from January 2014 to December 2015 in Shanghai First Maternity and Infant Hospital in Shanghai, China. Since HDP is generally clinically diagnosed beginning at 20 gestational weeks and we did not have detailed information on the diagnosis date of HDP, women with an initial visit at a gestational week before 20 weeks were included in the study (*n* = 21,944). Details of the study population have also been described previously [19].

All women were invited to provide personal sociodemographic and health information at the initial antenatal appointment, including age at conception, residence address, employment status (employed or unemployed), health insurance (with or without), parity (nulliparous or multiparous), height (in centimetres), and weight before pregnancy (in kilograms). The pre-conception body mass index (BMI) was calculated by dividing the weight (in kilograms) by the height (in metres squared) and was categorized into three groups according to WHO cut-off points recommendations for Asian women [20]: underweight (BMI < 18.5 kg m^− 2^), normal weight (BMI 18.5–23 kg m^− 2^), overweight (23–27.5 kg m^− 2^) or obese (BMI ≥ 27.5 kg m^− 2^). Gestational age was assessed based on the date of the last menstrual period and the results of the early ultrasound. We also asked women if either of their parents had a history of chronic diseases, including heart diseases, diabetes, hypertension, and kidney diseases.

Women with missing or implausible pre-pregnancy weight or weight at initial clinic (< 34 or > 150 kg), or height (< 100 cm) were excluded (*n* = 13,157). In addition, women with a diagnosis of chronic hypertension before pregnancy were also excluded from the study (*n* = 11). Finally, 8776 women were included in the study. We also analysed the difference of basic characteristic between included women and excluded women. Compared to the excluded women, the included women were more likely to have government-sponsored health care insurance. Other characteristics, including age, gestational age, sex of foetus, season of conception are comparable between the two groups (Table S1 in additional file).

### Fine particulate matter (PM_2.5_) exposure assessment

A land-use regression (LUR) model, a feasible way to describe the relationship between land use and PM_2.5_ pollution level, was used to predict outdoor PM_2.5_ levels at the residential address of each participating woman [21, 22]. The dependent variable in LUR model was the mean values of PM_2.5_ concentration collected by Shanghai Environmental Monitor Centre at 35 monitoring locations. The independent variables included longitude, distance from monitors to the ocean, highway intensity, waterbody area, and industrial land area. Forward stepwise multiple regression method was employed to fit the model. The adjusted R squared value for the model was 0.88. This method was described in more detail by C Liu et al. 2016 [21]. A map showing the locations of participants relative to the monitors is provided in our previous article [23]. We defined a priori three exposure windows of PM_2.5_ according to the birthdate and gestation weeks in this study, including the first trimester (0–12 gestational weeks), early second trimester (13–20 gestational weeks) and the period before a potential diagnosis of HDP (0–20 gestational weeks). We log-transformed the PM_2.5_ concentrations measured in the different gestational periods to improve normality and variance homogeneity [24]. Concentrations of PM_2.5_ were analysed as continuous or categorical variables according to the interquartile range (IQR) in the study.

### Outcome

The outcome of the study was HDP. Women with an onset of hypertension (systolic blood pressure and/or diastolic blood pressure ≥ 140/90 mmHg) after 20 weeks of gestation were diagnosed with HDP during the study period (2014–2015). Information on HDP was identified from electronic medical records and the maternal hospital discharge summary by an information engineer. Two trained researchers double-checked the information by referring to scanned medical records. In our main analysis, we combined gestational hypertension, pre-eclampsia, eclampsia, and hemolytic anemia, elevated liver function and low platelet count syndrome (HELLP syndrome) as a whole group.

### Statistical analysis

Descriptive statistics for participant characteristics are presented as the means [standard deviations (SD)] for continuous variables and frequencies with percentages for categorical variables. The distribution of PM_2.5_ exposure was presented as medians and IQR during each of the specified time windows.

Firstly, we assessed the possibility of a nonlinear relationship between PM_2.5_ exposure and the risk of HDP using Poisson regression model with restricted cubic splines (RCS) with 3 knots (the 25th, 50th, 75th percentile) for PM_2.5_ [25]. The 50th percentile of the PM_2.5_ was treated as reference. As the results did not indicate a nonlinear association between PM_2.5_ and risk of HDP, the relative risks (RR) and 95% confidence interval (CI) for PM_2.5_ exposure during the index gestational period and the risk of HDP was estimated through Poisson regression analysis without RCS by PROC GENMOD in SAS 9.4 in the main analysis. The RR per 10 μg m^− 3^ or per IQR and 95% CI for an increase in the PM_2.5_ concentration during a specific gestational period were obtained. Covariates in the adjusted multivariable analysis were identified a priori based on association with both PM_2.5_ and HDP. We included maternal age, parity, parental history of chronic diseases, health insurance, sex of foetus, and season of conception [17, 26, 27]. Considering the different pathophysiology for subtypes of hypertension, we reran the analyses for gestational hypertension and preeclampsia separately.

In a sensitivity analysis, we performed exploratory analysis to explore other covariates that are known to or might modify the association between PM_2.5_ exposure during the first trimester and the risk of HDP on the additive and multiplicative scales, including maternal age (< 35 or ≥ 35 years), pre-conception BMI (underweight, normal weight, overweight or obese), parental history of hypertension (yes, no), season of conception (spring and winter, summer and autumn), and parity (nulliparous or multiparous). The multiplicative interaction was evaluated by including the interaction index in the models. The additive interaction was evaluated by the relative excess risk due to the interaction (RERI) [28]. If an additive interaction was not observed, the 95% CI of the RERI would include zero.

All statistical tests were two-sided and used an α level of 0.05. Statistical analyses were performed using SAS 9.4 (SAS Institute Inc., Cary, NC, USA). Figures were prepared with Stata/SE15 (StataCorp, College Station, TX, USA).

## Results

Of the 8776 women in the study population, 440 (5.01%) women were diagnosed with HDP. Table 1 shows the demographic characteristics of the participants. At baseline, the mean age of the participants was 30.1 (SD = 3.6) years at conception, 7117 (81.1%) were nulliparous, 1659 (18.9%) were multiparous, 1436 (16.4%) were overweight, 226 (2.6%) were obese, and 1408 (16.0%) were underweight. Notably, 3136 (35.7%) women became pregnant in the spring, 1778 (20.3%) in the summer, 1541 (17.6%) in the autumn and 2321 (26.5%) in the winter. Greater than 30 % of women (*n* = 2749) reported a history of chronic diseases in either of their parents. Compared to normotensive women, women with HDP tended to be overweight or obese and were more likely to be nulliparous or have a parental history of chronic diseases.

**Table 1:** Basic Characteristics of the participants (*n* = 8776)

Characteristics	Total (*n* = 8776)	No HDP (*n* = 8336)	HDP (*n* = 440)	*P* value
Gestational age at enrollment (days)	104.0 **±** 8.7	104.0 **±** 8.6	104.3 **±** 8.9	0.46
Maternal age at enrollment (year)	30.2 **±** 3.6	30.2 **±** 3.6	30.4 **±** 3.7	0.14
Maternal height (centimeters)	161.7 **±** 4.8	161.7 **±** 4.8	161.7 **±** 5.0	0.85
Maternal weight before pregnancy (kg)	54.8 **±** 7.8	54.6 **±** 7.5	59.8 **±** 10.9	< 0.01
Pre - conception BMI (kg m^− 2^)				
Underweight (< 18.5)	1408 (16.0)	1372 (16.5)	36 (8.2)	
Normal (18.5–23)	5706 (65.0)	5486 (73.6)	220 (50.0)	
Overweight (23–27.5)	1436 (16.4)	1298 (15.6)	138 (31.4)	
Obesity (≥27.5)	226 (2.6)	180 (2.2)	46 (10.5)	< 0.01
Season of conception				
Spring	3136 (35.7)	2951 (35.4)	185 (42.1)	
Summer	1778 (20.3)	1695 (20.3)	83 (18.9)	
Autumn	1541 (17.6)	1492 (17.9)	49 (11.1)	
Winter	2321 (26.5)	2198 (26.4)	123 (27.9)	< 0.01
Parity				
Nulliparous	7117 (81.1)	6735 (80.8)	382 (86.8)	
Multiparous	1659 (18.9)	1601 (19.2)	58 (13.2)	< 0.01
Sex of foetus				
Male	4510 (51.4)	4284 (51.4)	226 (51.4)	
Female	4266 (48.6)	4085 (48.6)	214 (48.6)	0.99
Parental history of chronic diseases				
No	6027 (68.7)	5777 (69.3)	250 (56.8)	
Yes	2749 (31.3)	2559 (30.7)	190 (43.2)	< 0.01
Prenatal care insurance type				
Government-sponsored	6900 (78.6)	6535 (78.4)	365 (83.0)	
Self-pay	1876 (21.4)	1801 (21.6)	75 (17.0)	0.02

Table 2 shows the average concentration to PM_2.5_ exposure among the study participants in different exposure periods. The PM_2.5_ concentration measured throughout the 20-week period ranged from 28.6 to 74.8 μg m^−3^ (median, 51.4 μg m^− 3^; IQR: 47.3–57.8 μg m^− 3^). Median (IQR) PM_2.5_ concentrations recorded in the first and second trimester of pregnancy were 54.6 μg m^− 3^ (IQR: 46.5–60.1 μg m^− 3^) and 52.0 μg m^− 3^ (IQR: 44.3–56.3 μg m^− 3^), respectively. The PM_2.5_ concentration is higher among women with HDP compared to women without HDP in first trimester and the whole 0–20 gestational weeks (*P* < 0.05).

**Table 2:** Summary of estimated PM2.5 concentration (μg m^− 3^)

Gestational period	Median (Q1 - Q3)			*P* value
	Total	No HDP	HDP	
0–12 gestational weeks	54.6 (46.5–60.1)	54.4 (46.3–60.0)	57.2 (50.1–60.6)	< 0.01
13–20 gestational weeks	52.0 (44.3–56.3)	52.0 (44.3–56.3)	52.1 (44.2–56.5)	0.68
0–20 gestational weeks	51.4 (47.3–57.8)	51.3 (47.3–57.8)	54.1 (47.8–58.3)	0.03

**Fig. 1:**
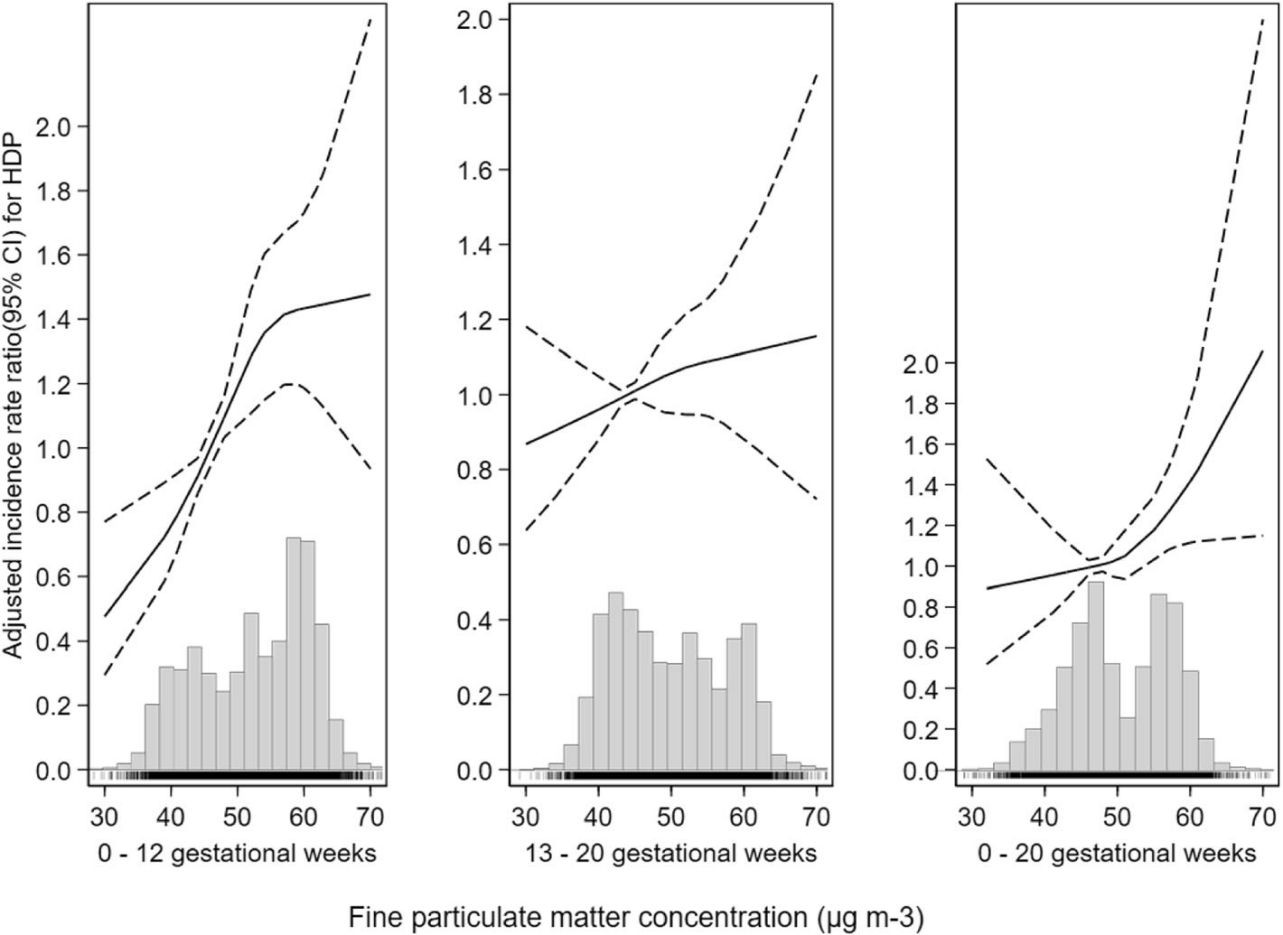
The curve shows nonlinear exposure - response association between trimester exposure of PM_2.5_ and adjusted RR (95% CI) for HDP. The histogram at the bottom shows the relative overall distribution of PM_2.5_ concentrations in the different period. The rug plot shows the amount of women according the PM_2.5_ concentration

The restricted cubic spline of the exposure-response relationship between PM_2.5_ exposure at different gestational periods and the risk of HDP was illustrated in Fig. 1). After adjusting for maternal age, parity, parental history of chronical diseases, health insurance, sex of foetus, and season of conception, the RR for HDP increased monotonically following an increase in the PM_2.5_ concentration in a positive exposure-response relationship pattern for each exposure period examined, which indicated a linear association between them.

We observed an increased risk of HDP associated with PM_2.5_ exposure during the first trimester, but not during the second trimester and or the entire 0–20 gestational week period (Table 3). The adjusted RR was 3.89 per 10 μg m^− 3^ (95% CI: 1.45–10.43) for the first trimester, 0.61 per 10 μg m^− 3^ (95% CI, 0.33–1.11) for the second trimester, and 0.71 per 10 μg m^− 3^ (95% CI, 0.40–1.27) for the entire 0–20 gestational week period.

**Table 3:** Association of PM_2.5_ and HDP by gestational periods

	No. of cases	(%)	Crude RR (95% CI)	Adjusted RR (95% CI)	*P* for trend
**0–12 gestational weeks**					
Continuous	440	5.1	4.03 (2.11–7.72)	3.89 (1.45–10.43)	
Q1 (< 46.36)	76	3.5	Reference (1.00)	Reference (1.00)	
Q2 (46.36–54.54)	103	4.7	1.33 (0.99–1.79)	1.30 (0.93–1.82)	
Q3 (54.54–60.06)	129	5.9	1.67 (1.26–2.22)	1.66 (1.11–2.48)	
Q4 (≥ 60.06)	132	6.0	1.70 (1.28–2.26)	1.67 (1.10–2.53)	< 0.01
**13–20 gestational weeks**					
Continuous	440	5.0	1.11 (0.66–1.87)	0.71 (0.40–1.27)	
Q1 (< 44.41)	113	5.1	Reference (1.00)	Reference (1.00)	
Q2 (44.41–52.03)	105	4.8	0.93 (0.71–1.21)	0.85 (0.65–1.12)	
Q3 (52.03–56.35)	109	5.0	0.98 (0.75–1.27)	0.88 (0.67–1.15)	
Q4 (≥ 56.35)	113	5.2	1.02 (0.78–1.32)	0.85 (0.64–1.12)	0.82
**0–20 gestational weeks**					
Continuous	440	5.0	2.22 (1.05–4.68)	1.00 (0.38–2.61)	
Q1 (< 47.35)	97	4.4	Reference (1.00)	Reference (1.00)	
Q2 (47.35–51.42)	103	4.7	1.06 (0.80–1.40)	0.90 (0.67–1.23)	
Q3 (51.42–57.81)	111	5.1	1.14 (0.87–1.50)	0.89 (0.65–1.24)	
Q4 (≥ 57.81)	129	5.9	1.33 (1.03–1.74)	1.01 (0.73–1.41)	0.02

When the exposure was categorized into four groups, participants exposed to the second, third and fourth quartiles of PM_2.5_ had RR of 1.30 (95% CI: 0.93–1.82), 1.66 (95% CI: 1.11–2.48) and 1.67 (95% CI: 1.10–2.53), respectively, for HDP compared with participants exposed to the first quartile of ambient PM_2.5_ concentrations. The *P*-value for the Cochran-Armitage trend test was < 0.01. The association pattern of PM_2.5_ with preeclampsia or gestational hypertension was similar to the main analysis (data available upon request).

No multiplicative or additive interactions were observed between maternal age, pre-conception BMI, parental history of hypertension, season of delivery and PM_2.5_ concentration in the first trimester on the risk of HDP (Table 4). An additive interaction and multiplicative interaction between PM_2.5_ exposure and parity were detected, which indicated that parity modified the effect of PM_2.5_ exposure on the risk of HDP.

**Table 4:** Interaction of covariates and PM2.5 exposure during first trimester with the risk of HDP

Covariates	Multiplicative scale	Additive scale
	RR (95% CI)	RERI (95% CI)^a^
Maternal age (≥ 35 years)	0.61 (0.33–1.15)	0.32 (− 0.18–0.81)
Parity (Nulliparous)	2.28 (1.28–4.06)	0.92 (0.46–1.38)
Parental history of chronical diseases (Yes)	1.04 (0.69–1.55)	0.20 (− 1.22–1.62)
Season of conception (Spring or winter)	0.89 (0.31–1.92)	−0.09 (− 1.62–1.44)
Pre-conception BMI (Underweight)	2.03 (0.93–4.46)	0.42 (− 0.10–0.93)
Pre-conception BMI (Overweight or obesity)	1.24 (0.81–1.87)	0.26 (−0.47–0.99)

^a^RERI = 0: indicates no interaction. RERI < 0: indicates negative interaction or less than additive interaction. RERI > 0: indicates positive interaction or more than additive interaction

## Discussion

We estimate the association between PM_2.5_ exposure during specific gestational periods and risk of HDP in a retrospective hospital-based cohort study. Based on the results, pregnant women might be at higher risk of developing HDP following exposure to PM_2.5_ in the first trimester. Furthermore, the association shows a positive exposure-response pattern and is modified by parity.

We observed a prevalence of 5% for HDP in this cohort, which is consistent with findings that reported a prevalence of 4.2% for HDP in China [29]. Our results were supported by a study which suggested that exposure to high levels of PM_2.5_ during early pregnancy was associated with increased prevalence of HDP [12]. Actually, the concentration of the PM_2.5_ in their study (median = 9.5 μg m^− 3^) is still much lower than the concentration in the present study (median = 54.1 μg m^− 3^); and the risk of HDP increased by 43% (from 24 to 67%) as per IQR increase of PM_2.5_ concentration. While another study, linking birth certificates and Community Air Survey data in New York city, did not provide clear evidence of an effect of ambient air pollution on HDP [10]. The author also stated that their results might be bias to the null due to the uncertain impact of adjusting for delivery hospital. In addition, the different models for estimating exposure made the data incomparable.

Although air quality in Shanghai is better than other large cities in China and the government has taken strategy to control air pollution since 2013 [30], the level of PM_2.5_ in this study was still higher than reported in previous studies performed in other countries. Dadvand et al. used LUR model to predict the PM_2.5_ concentration in 8398 women and reported that first and third trimester exposure to PM_2.5_ is associated with the risk of preeclampsia [31]. Mobasher et al. also reported a positive association between PM_2.5_ exposure and HDP during the first trimester and throughout pregnancy [16]. These findings are consistent with our results on the elevated risk of HDP following PM_2.5_ exposure in the first trimester. The average PM_2.5_ concentrations reported in their studies (16.5 and 18.1 μg m^− 3^, respectively) are much lower than the concentration recorded in our study (51.4 μg m^− 3^).

Few studies have tested the interaction effect of demographic characteristics and PM_2.5_ exposure on the HDP. According to Mobasher et al., the association between PM_2.5_ and HDP is modified by BMI. Kannan et al. also reported a positive association between PM_2.5_ exposure and increased pulse pressure for participants categorized as obese (BMI ≥ 30 kg m^− 2^) [32]. Due to the ethnic differences between American women and Asian women, the criteria used to establish BMI categories and the prevalence for overweight or obesity are different in the two studies. We identified a modifying effect of parity instead of pre-conception BMI. The association is more obvious for the nulliparous women. The childbearing policy also leads to a different distribution in parity among western countries and China. Specifically, more nulliparous women were included in our study (81% vs. 56%). A previous study performed among Asian-American women showed that nulliparity is significantly associated with gestational hypertension [33]. Another study involving 49 hospitals in Canada also reported that a history of term pregnancy conveys a substantial “protection” against preeclampsia [34]. Given the high proportion of nullipara in our study, further studies are needed to confirm our findings on parity.

The potential mechanisms underlying the associations between PM_2.5_ exposure and HDP remain unclear. However, first trimester trophoblast cells that are exposed to PM_2.5_ respond with reduced growth, oxidative stress, inflammation and endoplasmic reticulum stress [35, 36]. In addition, an animal study observed the induction of a persistent intrauterine inflammatory state following PM_2.5_ exposure, and the greatest effect was observed for the first trimester exposure [37]. Histopathological changes and vascular injuries of the placenta were also observed in mice exposed to PM_2.5_ [38]. All these changes were reported to underlie the pathogenesis of HDP [39].

To our knowledge, this study represents the first attempt to estimate the association between exposure to high concentrations of PM_2.5_ and its interaction with demographic characteristics with HDP in the Chinese population, which consolidated the evidence on the association of PM_2.5_ with hypertension. While some limitations should also be noted. First, although the baseline characteristics are comparable between the included women and excluded women, selection bias cannot be ruled out and further study is needed to consolidate the findings in our study. In addition, single geographic region of Shanghai limits the generalizability of our finds. Second, the exact blood pressure and diagnosis date of HDP were unavailable, which might result in exposure misclassification. Women with higher exposure during the late second trimester but not in the first trimester would be classified as unexposed and bias the result towards the null. Meanwhile, the estimates were robust when we included or excluded the PM_2.5_ exposure in the late period of the second trimester (20–28 gestational weeks, data available upon request). Third, the deidentified electronic medical system does not have data on lifestyle factors (maternal smoking, alcohol use), diet, and other potential risk factors for HDP that might induce residual confounding. Although smoking might be a strong effect modified factor in this study, the proportion of active smoking is rare among Chinese pregnant women. Meanwhile, the passive smoking rate of pregnant women also sharply decreased to 7.8% consequently since public places smoking control regulations issued in 2010 [40]. Our data also suggested that the included women were more likely to have government-sponsored health care insurance, indicating a higher social status, less likely to be a smoker, and more likely to lead a healthy lifestyle. Hence, adjusting for health insurance in the regression model partly reduced the confounding related to the lifestyle. Furthermore, a case-control study of approximately 2500 births in Los Angeles County indicated that associations between air pollution and preterm birth were insensitive to adjustment for occupation or income [41]. Finally, although our cohort was compiled during 2014–2015 in a single institute and no change of definition of HDP were warrants, the lack of specific diagnosis might result in misclassification of HDP. We believe that the misclassification is nondifferential as it is unlikely to be related the PM_2.5_ exposure, thus bias the estimates towards the null again.

## Conclusion

In conclusion, exposure to PM_2.5_ during the first trimester tends to be associated with an increased risk of HDP. The effect estimates are more obvious for nulliparous women than multiparous women. Our findings suggested that the concentration of PM_2.5_ is still high in Shanghai, China. There is a need for better and sustainable air pollution control in order to reduce the disease burden of HDP among pregnant women, especially for nulliparous women.

## Supplementary information

**Additional file 1: Table S1.** Comparison of basic characteristic between included and excluded women.

## Data Availability

The data that support the findings of this study are available from Shanghai First maternity and Infant hospital but restrictions apply to the availability of these data, which were used under license for the current study, and so are not publicly available. Data are however available from the authors upon reasonable request and with permission of Shanghai First maternity and Infant hospital.

## Abbreviations

PM_2.5_: Fine particulate matter
HDP: Hypertensive disorders of pregnancy
IQR: Interquartile range
BMI: Body mass index
LUR: Land-use regression
SD: Standard deviations
RR: Relative risk
CI: Confidence interval
