# Supplementary material for: Correlation between exposure to fine particulate matter and hypertensive disorders of pregnancy in Shanghai, China
Source: Environ Health. 2020 Sep 17;19:101. doi: 10.1186/s12940-020-00655-1 (PMC7499904; doi:10.1186/s12940-020-00655-1)
Supplement: Supplementary file 1 — Additional file 1: Table S1. Comparison of basic characteristic between included and excluded women. [file 12940_2020_655_MOESM1_ESM.docx]

Table S1: Comparison of basic characteristic between included and excluded women.

| Characteristic | Included women | Excluded women |
| --- | --- | --- |
| Age | 30.1±3.6 | 31.0±3.9 |
| Gestational age | 275±9 | 275±10 |
| Height | 161.7±4.8 | 162.1±4.9 |
| Season of conception |  |  |
| Spring | 3,136 (35.7) | 4,877 (37.0) |
| Summer | 1,778 (20.3) | 2,715 (20.6) |
| Autumn | 1,541 (17.6) | 1,849 (14.0) |
| Winter | 2,321 (26.5) | 3,727 (28.3) |
| Parity |  |  |
| Nulliparous | 7,117 (81.1) | 9,815 (74.6) |
| Multiparous | 1,659 (18.9) | 3,350 (25.4) |
| Baby sex |  |  |
| Male | 4,510 (51.4) | 6,813(51.7) |
| Female | 4,266 (48.6) | 6,355 (48.2) |
| Prenatal care insurance type |  |  |
| Government-sponsored | 6,900 (78.6) | 6,534 (49.6) |
| Self-pay | 1,876 (21.4) | 6,634(50.4) |
